# Prevalence and Characteristics of Metabolic Dysfunction-Associated Fatty Liver Disease among an Iranian Adult Population with Ethnic and Genetic Diversity: Results of the PolyIran-Liver Study

**DOI:** 10.34172/mejdd.2024.374

**Published:** 2024-04-30

**Authors:** Elham Jafari, Shahin Merat, Amir Anoushiravani, Amir Reza Radmard, Gholamreza Roshandel, Maryam Sharafkhah, Masoud Khoshnia, Alireza Nateghi, Abolfazl Shiravi Khuzani, Hossein Poustchi, Reza Malekzadeh

**Affiliations:** ^1^Liver and Pancreatobiliary Diseases Research Center, Digestive Diseases Research Institute, Tehran University of Medical Sciences, Tehran, Iran; ^2^Digestive Oncology Research Center, Digestive Disease Research Institute, Tehran University of Medical Sciences, Tehran, Iran; ^3^Digestive Disease Research Center, Digestive Disease Research Institute, Tehran University of Medical Sciences, Tehran, Iran; ^4^Department of Radiology, Shariati Hospital, Tehran University of Medical Sciences, Tehran, Iran; ^5^Golestan Research Center of Gastroenterology and Hepatology, Golestan University of Medical Sciences, Gorgan, Iran; ^6^Research and Development Department, Alborz-Darou Pharmaceutical Co., Ghazvin, Iran

**Keywords:** MAFLD, NAFLD, Ethnicity, Genetic, Insulin resistance, Iran

## Abstract

**Background:**

Metabolic dysfunction-associated fatty liver disease (MAFLD) is a rising global public health concern. It has been demonstrated that its prevalence and characteristics vary by region and racial/ethnicity. We aimed to investigate the prevalence of MAFLD and its characteristics among Turkmen and non-Turkmen ethnic groups in a multiethnic population region of Iran.

**Methods:**

In this cross-sectional study, we analyzed baseline data for 1614 participants, aged above 50 years, from the PolyIran-Liver trial who were randomly selected from Gonabad city and determined the prevalence of MAFLD and its demographic and metabolic disorders for both the Turkmen and non-Turkmen ethnic groups. Multivariate binary logistic regressions were applied to identify MAFLD-associated factors for men and women separately for the Turkmen and non-Turkmen populations.

**Results:**

The mean (SD) age of the participants was 59.1(6.7) years. Of the participants, 51.5% (n=831) were men, and 52.9% (n=854) were Turkmen. The prevalence of MAFLD among the overall study population was 39.8% (n=614). It was more common among women (45.8% vs. 34.1% in men, *P*<0.001), non-Turkmens (43.9% vs. 36.1% in Turkmens, *P*<0.001), and at age 50-64 (41.5% vs.36.1% in age≥65 *P*=0.004). The fully adjusted multivariate analysis in sex strata exhibited an independent negative association between Turkmen ethnicity only among men but not among women. The increased waist circumference (WC) was the most common metabolic disorder, observed in more than 95.5% of patients with MAFLD (*P*<0.001). Multivariate analysis in sex/ethnic strata with adjustment for potential confounders revealed an independent association of MAFLD with increased WC, insulin resistance, impaired fasting glucose/diabetes type 2, and high alanine aminotransferase (ALT) among women in both ethnic groups while with elevated triglyceride (TG) only among Turkmen and high body mass index (BMI) only among non-Turkmen women. Increased WC had the strongest independent association with MAFLD among women and the highest odds ratio (OR) with MAFLD in Turkmen women (OR: 6.10; 95% CI 1.56-23.86 vs. 4.80 in non-Turkmen women). Among men, MAFLD was independently associated with insulin resistance, high BMI, and high ALT in both ethnic groups and elevated TG only in non-Turkmen men (all *P*<0.001). Insulin resistance had the strongest independent OR with MAFLD among men with similar size in both ethnic groups (4.68 [95% CI 2.56-8.55]) in non-Turkmen men and 4.37 (95% CI 2.27-8.42 in Turkmen men).

**Conclusion:**

This study revealed the high prevalence of MAFLD with a sex and ethnic disparity in the middle-aged population of Gonabad city. Further research is needed to understand the factors contributing to the higher prevalence of MAFLD in this region, particularly in women. Furthermore, considering the diverse ethnic population of Iran, it is suggested that future investigations on the sex and ethnic aspects of MAFLD in the Iranian population be conducted to provide targeted prevention strategies better suited for the Iranian population.

## Introduction

 Metabolic dysfunction-associated fatty liver disease (MAFLD) is the most common chronic liver disease, affecting one-third of the Middle East and one-fourth of the population globally.^1,2^

 MAFLD is a new concept of fatty liver disease defined by the presence of hepatic steatosis and having at least one of the metabolic features, such as overweight or obesity, type 2 diabetes mellitus (T2DM), or metabolic dysregulation.^3^ Patients with MAFLD are more than metabolically normal non-alcoholic fatty liver disease (NAFLD) at risk of progression of liver damage, cardiovascular events, and all-cause mortality, which makes it a major public health concern.^4-7^

 The global prevalence of NAFLD/MAFLD is 20%-25% of the adult population. Currently, there is no proven drug to cure MAFLD. Modifying lifestyle and managing risk factors and metabolic morbidities are essential to improve complications and consequences. Obesity, insulin resistance, and diabetes are the most well-known metabolic risk factors that must be controlled in different populations.^8-11^ Genetic factors also have a significant role in the development and progression of NAFLD/MAFLD. Different ethnicities have their specific lifestyle and nutrition habits and variations in the genetic predisposition to NAFLD and its associated complications and metabolic disorders (MAFLD). Accordingly, the prevalence of NAFLD/MAFLD varies among diverse racial/ethnic populations, which causes the disease to exhibit a distinct metabolic profile across various racial or ethnic groups.^2,12,13^

 Therefore, identifying specific characteristics of NAFLD/MAFLD and its associated complications within each region and specific ethnic population is crucial for accurate diagnosis, prevention strategies, appropriate management, and improving patient outcomes. Several studies have investigated the ethnic diversity of NAFLD/MAFLD and its metabolic features, including insulin resistance, dyslipidemia, hypertension, and obesity patterns, in the respective countries.^14-17^

 The Middle East is among the regions with the highest and increasing prevalence of NAFLD/MAFLD. It is assumed that this might be due to the greater susceptibility of some ethnic groups to NAFLD/MAFLD in this region, and some ethnic and genetic predisposing factors. In addition, socioeconomic factors and lifestyle habits play a role in this high prevalence and its complications.^2^

 Iran is a Middle Eastern country with a multiethnic population. However, this issue has not been investigated, and there is no available information on the specific characteristics of NAFLD/MAFLD and its metabolic profile among the ethnic groups of Iranians.

 Therefore, we investigated the prevalence of MAFLD and its metabolic disorders among various ethnic groups in a middle-aged population with a diverse ethnic composition predominantly consisting of Turkmen.

## Methods and Methods

###  Study Population 

 We conducted this cross-sectional study on the baseline data of the PolyIran-Liver study. The PolyIran-Liver study is a nested randomized controlled trial in the Golestan Cohort Study (GCS) carried out from September 2011 to March 2014.^18^ The GCS is a population-based prospective cohort study launched in 2004 with 50045 participants aged 40-75 years residing in the Golestan province, northern Iran.^19^

 A random sample of 2500 urban participants of the GCS who resided in Gonabad, aged 50 and older, were selected for the PolyIran-Liver study. After the exclusion of participants with viral or autoimmune hepatitis or hepatitis caused by drugs and other exclusion criteria, a total of 1614 consented and underwent ultrasonography for assessment of NAFLD. Individuals who consented to attend the trial underwent liver elastography as well.

 The data collection and sampling methods are presented in detail in the PolyIran-Liver protocol.^18^ In short, all participants were interviewed, and the baseline information, demographic factors, smoking, using opium, alcohol drinking, and physical activity were collected using a lifestyle questionnaire. Trained technicians measured the anthropometric data and collected the blood samples from the participants after the interview.

###  Variable and Definitions

 Physical activity was defined as yes or no according to the WHO’s recommendations for physical activity for adults. Ever smokers or opium users were defined as those who had used tobacco or opium at least once a week for more than 6 months. An alcohol drinker was defined as those who had mild levels of alcohol consumption at least once a week for more than six months. The poverty level was defined as the lowest tertile of wealth score composition, calculated based on ownership of household appliances, vehicles, and other variables associated with wealth, using multiple correspondence analyses. Body mass index (BMI) was calculated by dividing measured weight (kg) by the square of the measured height (m), and overweight/obesity was considered as BMI ≥ 25 kg/m^2^.^20^ Increased waist circumference was considered as WC ≥ 90.^21^ Having metabolic syndrome (MetS) was defined based on the Third Report of the National Cholesterol Education Program (NCEP ATP III) as the presence of at least three of the following metabolic abnormalities: WC ≥ 90, blood pressure ≥ 130/85 mm Hg or specific drug treatment; fasting triglyceride (TG) level ≥ 150 mg/dL or specific drug treatment; fasting plasma HDL-C < 40 mg/dL for men and < 50 mg/dL for women or specific drug treatment; fasting blood glucose ≥ 100 (mg/dL) or specific drug treatment.^22^ Insulin resistance was considered as homeostatic model assessment for insulin resistance (HOMA-IR) > 2.5, high alanine aminotransferase (ALT) was defined as levels above 45 and 30 IU/L for men and women, respectively, and liver stiffness measurement ≥ 7 was considered to be high liver fibrosis.

###  Outcome Assessment

 MAFLD was considered as an outcome and was diagnosed based on ultrasound-diagnosed hepatic steatosis, in addition to one of the following three criteria: overweight/obesity BMI ≥ 25 kg/m^2^, presence of T2DM, or evidence of metabolic dysregulation in lean individuals.

 The metabolic dysregulation was defined as the presence of at least two following metabolic risk abnormalities: WC ≥ 90 (21 ); blood pressure ≥ 130/85 mm Hg or specific drug treatment; fasting TG level ≥ 150 mg/dL or specific drug treatment; fasting plasma HDL-C < 40 mg/dL for men and < 50 mg/dL for women or specific drug treatment; impaired fasting glucose (fasting blood glucose between 100 to 125 mg/dL, and self-report has not been clearly diagnosed as diabetes), HOMA-IR > 2.5.^3^

###  Ultrasound Evaluation

 Ultrasound assessment was performed using an Accuvix XQ ultrasound unit (Medison, Seoul, Korea) equipped with a 3-7 MHz curved-array and a 5-12 MHz linear-array transducer to evaluate liver, abdominal fat and carotid arteries. The presence of hepatic steatosis was determined using the ultrasonographic scoring system, with high specificity (100%) and sensitivity (91.7%) for the histological diagnosis of fatty liver.^23^

 Ultrasonographic findings were scored according to the protocol; hepatorenal echo contrast and/or liver brightness (0 to 3), deep attenuation (0 to 2), and vascular blurring (0 to 1) were used for scoring. A total score of at least 2, which includes the hepatorenal echo contrast and/or bright liver score of at least 1 was defined as NAFLD.^18,24^

###  Transient Elastography

 Liver stiffness was measured by transient elastography using the FibroScan® 502 (EchoSense, Paris, France, 5 MHz). According to the manufacturer’s guidelines, the M probe was used for participants with a thoracic perimeter of less than 110 cm and the XL probe for 110 cm and above. With the patient lying in the dorsal decubitus position with maximal abduction of the right arm, the probe was placed on the patient’s skin, overlying the right lobe of the liver through the intercostal spaces. At least 10 measurements were done for each patient, and the median value was recorded. Values were considered valid if the interquartile range (IQR) was less than 30% of the median reading and the success rate was at least 60%.^18^

###  Statistics Analysis

 We estimated the prevalence of MAFLD for the overall study population. Bivariate analysis, using the chi-square test, was conducted to determine statistical differences between the categorical variables in the prevalence of MAFLD. For the comparison of quantitative variables among study arms, a two-sample independent t-test or non-parametric Mann-Whitney U test was used, as appropriate. Multivariate binary logistic regression analysis was performed, adjusting for statistically significant variables identified in the bivariate analysis as confounding factors to determine the independent factors associated with MAFLD in ethnic and sex groups. To examine the role of sex in the relationship between ethnicity and MAFLD, we conducted a stratified logistic regression analysis, adjusting for the significant variables separately for males and females. The data are presented as adjusted odds ratios (ORs) with their corresponding 95% confidence intervals. A *P *value of < 0.05 was considered statistically significant. The analyses were carried out using SPSS software version 22.0.

## Results

 Of the 1614 participants, 80.1% were aged 50-64, while 19.9% were 65 years and older. Among them, 52.9% belonged to the Turkmen ethnicity, and 51.5% were men. The overall prevalence of MAFLD was 39.8% (642 out of 1614 participants). The patients diagnosed with MAFLD had a mean age of 58.5 ± 6.3, which was lower compared with those without MAFLD (59.5 ± 6.8, *P* < 0.001). MAFLD was more prevalent in women than in men (45.8% vs. 34.1%, *P* < 0.001), non-Turkmens than Turkmens (43.9% vs. 36.1%, *P* < 0.001), and the age group below 65 compared with the older age group (41.5% vs. 36.1%, *P* = 0.004) (Table 1).

**Table 1:** Population characteristics by MAFLD status

	**Overall (n=1614)**	**MAFLD (n=642)**	**Non- MAFLD (n=972)**	***P*** ** value**
Age (Mean ± SD)	59.1 ± 6.7	58.5 ± 6.3	59.5 ± 6.8	0.001
Age group, % (n)				
Age < 65	80.1 (1293)	41.5 (537)	58.5 (756)	0.004
Age ≥ 65	19.1 (321	32.7 (105)	67.3 (216)	
Ethnicity, % (n)				
Turkmen	52.9 (854)	36.1 (308)	43.9 (426)	0.001
Non-Turkmen	47.1 (760)	43.9 (334)	56.1 (546)	
Sex, % (n)				
Male	51.5 (831)	34.1 (283)	65.9 (558)	< 0.001
Female	48.5 (783)	45.8 (359)	54.2 (424)	

###  The Baseline Demographic Characteristics of MAFLD by Ethnic Groups

 Since demographic factors can vary based on ethnic and cultural characteristics, we examined the prevalence of these factors separately for MAFLD and non-MAFLD participants in two distinct groups: Turkmen and non-Turkmen populations. As presented in Table 2, there were similarities and differences in the demographic characteristics of MAFLD patients between the two ethnic groups.

**Table 2:** The baseline demographic characteristics of MAFLD by ethnic groups

		**Turkmen (n=854)**			**Non-Turkmen (n=760)**		
	**Overall** **(n=1614)**	**MAFLD** **n=308**	**Non-MAFLD** **n=546**	**P value**	**MAFLD** **n=334**	**Non-MAFLD** **n=426**	**P value**
Age group % (n)							
Age < 65	80.1 (1290)	37.6 (255)	63.4 (423)	0.065	45.9 (282)	54.1(333)	0.029
Age ≥ 65	19.9 (321)	30.1 (53)	69.9 (123)		35.9 (52)	64.1(93)	
Sex, % (n)							
Male	51.5 (831)	28.6 (124)	71.4 (310)	< 0.001	40.1 (159)	59.9(238)	0.024
Female	48.5 (783)	43.8 (184)	56.2 (236)		48.2 (175)	51.8(188)	
Education, % (n)							
≤ Primary school	62.9 (1014)	42.7 (159)	57.3 (213)	< 0.001	44.0 (114)	56.0 (145)	0.973
≥ Middle school	37.1 (600)	31.0 (149)	69.0 (332)		36.1 (308)	63.9 (545)	
Poverty level, % (n)							
Lowest tertial	32.6 (525)	35.4 (115)	64.6 (210)	0.521	42.0 (84)	58.0 (116)	0.730
Higher	67.4 (1086)	36.6 (193)	63.4 (335)		44.6 (249)	55.4 (309)	
Marital status, % (n)							
Married	90.1 (1462)	35.8 (276)	64.2 (496)	0.503	43.9 (303)	56.1 (387)	0.974
Else	8.9 (149)	39.5 (32)	60.5 (49)		44.1 (30)	55.9 (38)	
Smoker, % (n)							
Ever used	11.4 (184)	25.0 (26)	75.0 (78)	0.012	23.8 (19)	76.3 (61)	< 0.001
Never used	88.6 (1425)	37.7 (282)	62.3 (466)		46.2 (313)	53.8 (364)	
Opium, % (n)							
Ever used	7.5 (121)	14.3 (8)	85.7 (48)	< 0.001	27.7 (18)	72.3 (47)	0.006
Never used	92.5 (1490)	37.6 (300)	62.4 (497)		45.5 (316)	54.5 (378)	
Alcohol, % (n)							
Ever used	10.5 (170)	24.5 (26)	75.5 (80)	0.008	53.1 (34)	46.9 (30)	0.125
Never used	89.5 (1442)	37.8 (282)	62.2 (465)		43.2 (300)	56.8 (395)	
Physical Activity, % (n)							
Yes	9.8 (134)	15.0 (9)	85.0 (51)	< 0.001	40.5 (30)	59.5 (44)	0.717
No	90.2 (1235)	38.2 (253)	61.8 (409)		42.8 (245)	57.2 (328)	

 In both ethnic groups, the prevalence of MAFLD was higher among women and participants below the age of 65. However, this difference was not statistically significant among the Turkmen population. MAFLD showed a statistically significant association with lower education levels and lack of physical activity in the Turkmen population but not among the non-Turkmen population.

 Furthermore, the prevalence of MAFLD was lower among participants who had a history of ever smoking, opium use, and mild alcohol consumption. These differences were statistically significant in both groups, except for alcohol in non-Turkmen participants.

###  Comparison of the Mean Values of Metabolic Factors between MAFLD and Non-MAFLD Participants in Each Ethnic Population

 As shown in Table 3, patients with MAFLD in both ethnic populations exhibited higher body composition indices, blood pressure, and serum levels of fasting blood sugar (FBS), insulin, HOMA-IR, hemoglobin A1c (HbA1C), TG, total cholesterol (TC), LDL-C, ALT, AST, and gamma-glutamyl transferase (GGT), as well as lower HDL-C levels compared with non-MAFLD participants and, all these differences were statistically significant (*P* < 0.001).

**Table 3:** Comparison of metabolic factors between MAFLD and non-MAFLD participants by ethnic groups

	**Turkmen (n=854)**			**Non-Turkmen(n=760)**		
**Factors** **(Mean±SD)**	**MAFLD** **n=308**	**Non-MAFLD** **n=546**	***P*** ** value**	**MAFLD** **n=334**	**Non-MAFLD** **n=426**	***P*** ** value**
Body composition						
BMI, kg/m^2^	30.83 ± 4.38	26.50 ± 4.49	< 0.001	30.83 ± 4.53	26.96 ± 4.55	< 0.001
Waist, cm	106.76 ± 10.37	95.66 ± 11.63	< 0.001	105.73 ± 9.97	95.30 ± 11.83	< 0.001
HC, cm	104.31 ± 8.09	99.70 ± 8.02	< 0.001	104.52 ± 9.44	99.39 ± 8.06	< 0.001
WHR	1.02 ± 0.07	0.96 ± 0.08	< 0.001	1.01 ± 0.06	0.96 ± 0.07	< 0.001
Blood pressure						
SBP, mm Hg	137.12 ± 22.85	131.01 ± 22.02	< 0.001	134.97 ± 19.72	131.90 ± 20.84	< 0.001
DBP, mm Hg	82.39 ± 10.99	79.49 ± 10.49	< 0.001	81.93 ± 10.01	79.98 ± 11.05	< 0.001
Blood parameters						
TC, mg/dL	218.92 ± 42.98	211.26 ± 42.15	< 0.001	215.53 ± 40.76	210.29 ± 40.63	< 0.001
HDL, mg/dL	58.32 ± 14.16	62.61 ± 15.41	< 0.001	54.30 ± 12.41	58.54 ± 14.27	< 0.001
LDL, mg/dL	127.44 ± 33.40	124.38 ± 34.37	< 0.001	124.93 ± 33.35	124.26 ± 34.14	< 0.001
TG, mg/dL	169.02 ± 95.41	123.86 ± 63.82	< 0.001	182.87 ± 98.44	138.85 ± 71.11	< 0.001
FBS, mg/dL	120.92 ± 52.93	99.03 ± 29.79	< 0.001	130.47 ± 58.99	105.19 ± 39.52	< 0.001
HBA1c, mg/L, (n = 411)	7.07 ± 2.47	7.05 ± 2.24	< 0.001	7.29 ± 2.25	6.64 ± 2.49	< 0.001
Insulin, mU/L	14.59 ± 9.64	9.29 ± 7.91	< 0.001	14.46 ± 8.06	8.83 ± 6.33	< 0.001
HOMA-IR	4.32 ± 3.34	2.33 ± 2.12	< 0.001	4.61 ± 3.21	2.34 ± 2.48	< 0.001
AST, IU/L	23.34 ± 10.99	21.25 ± 12.92	< 0.001	22.34 ± 10.63	19.97 ± 7.67	< 0.001
ALT, IU/L	28.55 ± 15.34	20.32 ± 16.77	< 0.001	28.50 ± 15.03	19.47 ± 10.13	< 0.001
GGT, IU/L	35.92 ± 27.88	32.06 ± 50.20	< 0.001	40.21 ± 48.12	27.76 ± 23.42	< 0.001
LSM, kPa	5.74 ± 5.41	4.57 ± 2.01	< 0.001	5.61 ± 3.04	4.87 ± 3.97	< 0.001

BMI: Body mass index, HC: Hip circumference, WHR: Waist-to-hip ratio, SBP: Systolic Blood Pressure, DBP: Diastolic Blood Pressure, TC: Total cholesterol, HDL: high-density lipoprotein cholesterol, LDL: low- density lipoprotein cholesterol, TG: Triglyceride, FBS: fasting blood sugar, HBA1c: hemoglobin A1c, HOMA-IR: Homeostatic Model Assessment for Insulin resistance, AST: Aspartate aminotransferase, ALT: Alanine aminotransferase, GGT: Gamma-glutamyl transferase, LSM: liver stiffness measurement.

###  The Prevalence of Metabolic Disorders and Comorbidities among MAFLD and Non-MAFLD Participants, Categorized by Ethnicity (Turkmen and Non-Turkmen) and Sex

 We determined the prevalence of metabolic disorders and comorbidities according to MAFLD in sex and ethnic strata, as presented in Table 4. The data revealed that among patients with MAFLD in both ethnic and sex groups, those with a WC ≥ 90 cm had the highest proportion (more than 95.5%). The prevalence of overweight/obesity was between 89.5% and 98.3% among patients with MAFLD, with the highest proportion among women and non-Turkmen population. Insulin resistance, defined as HOMA-IR > 2.5, had a prevalence of 74.5% among non-Turkmen individuals and between 68.3% and 71.3% in the Turkmen population. The highest proportion of patients with prediabetes/diabetes (39.1%-40.8%) was found among women, while Turkmen men had the lowest proportion (21.8%). High blood pressure, with a prevalence of over 72.8%, was a common metabolic disorder in the entire study population, with the highest difference between MAFLD and Non-MAFLD participants observed among Turkmen men, and statistically significant only among men.

**Table 4:** Comparison of the prevalence of metabolic dysregulations and comorbidities between MAFLD and Non-MAFLD participants in sex/ethnic groups

		**Men (n=831)**			**Women (n=783)**		
	**Overall**	**MAFLD (n=283)**	**Non- MAFLD (n=538)**	***P*** ** value**	**MAFLD (n=359)_**	**Non- MAFLD (n=424)**	***P*** ** value**
BMI group ≥ 25							
Turkmen	73.2 (625)	89.5 (111)	52.5 (162)	< 0.001	95.1 (175)	75.0 (177)	< 0.001
Non-Turkmen	78.3 (595)	93.1 (149)	57.1 (136)	< 0.001	98.3 (172)	73.9 (139)	< 0.001
BMI group < 25							
Turkmen	26.8 (229)	10.5 (13)	47.7 (148)	< 0.001	4.9 (9)	25.0 (59)	< 0.001
Non-Turkmen	21.7 (165)	6.9 (11)	42.9 (102)	< 0.001	1.7 (3)	26.1 (49)	< 0.001
Waist ≥ 90cm							
Turkmen	81.2 (693)	96.8 (120)	70.6 (218)	< 0.001	98.4 (181)	73.7 (174)	< 0.001
Non-Turkmen	81.0 (615)	95.6 (152)	68.9 (164)	< 0.001	96.6 (168)	69.7 (131)	< 0.001
IFG /T2DM							
Turkmen	18.1 (154)	21.8 (27)	8.7 (27)	< 0.001	39.1 (72)	11.9 (28)	< 0.001
Non-Turkmen	25.8 (196)	35.2 (56)	16.8 (40)	< 0.001	40.8 (71)	15.4 (29)	< 0.001
Homa IR > 2.5							
Turkmen	44.7 (375)	71.3 (87)	26.2 (80)	< 0.001	68.3 (125)	36.4 (83)	< 0.001
Non-Turkmen	49.2 (364)	74.8 (116)	27.3 (62)	< 0.001	74.6 (129)	30.8 (57)	< 0.001
HTN							
Turkmen	73.7 (629)	81.5 (101)	69.9 (216)	0.019	77.2 (142)	72.0 (170)	0.255
Non-Turkmen	72.8 (552)	78.0 (124)	67.1 (159)	0.023	75.9 (132)	72.9 (137)	0.515
Elevated TG							
Turkmen	34.7 (269)	48.0 (59)	26.2 (81)	< 0.001	53.3 (98)	24.6 (58)	< 0.001
Non-Turkmen	49.6 (376)	64.2 (102)	39.2 (93)	< 0.001	58.6 (102)	42.0 (79)	0.002
LOW HDL							
Turkmen	16.0 (137)	15.3 (19)	10.0 (31)	0.098	28.3 (52)	14.8 (35)	0.001
Non-Turkmen	25.7 (195)	22.1 (35)	15.1 (36)	0.064	38.3 (67)	30.3 (57)	0.110
MCVE							
Turkmen	14.5 (124)	13.7 (17)	15.2 (47)	0.756	14.1 (26)	14.4 (34)	0.989
Non-Turkmen	18.9 (621)	20.1 (32)	19.8 (47)	0.861	18.4 (32)	13.8 (26)	0.237
MetS							
Turkmen	37.0 (316)	51.6 (64)	23.2 (72)	< 0.001	60.9 (112)	28.8 (68)	< 0.001
Non-Turkmen	50.0 (380)	64.2 (102)	36.1 (86)	< 0.001	66.3 (116)	40.4 (76)	< 0.001
High ALT							
Turkmen	30.5 (260)	44.4 (55)	12.3 (38)	< 0.001	56.5 (104)	26.7 (63)	< 0.001
Non-Turkmen	34.3 (260)	43.4 (69)	12.7 (30)	< 0.001	56.9 (99)	33.0 (62)	< 0.001
LSM ≥ 7							
Turkmen	9.3 (73)	17.9 (20)	7.2 (21)	0.002	10.9 (18)	6.5 (14)	0.123
Non-Turkmen	10.1 (69)	12.4 (18)	4.2 (9)	< 0.001	18.7 (28)	8.2 (14)	< 0.001

BMI: Body mass index, IFG/T2DM: impaired fasting glucose or type 2 diabetes mellitus, HOMA-IR: Homeostatic Model Assessment for Insulin resistance, HTN: hypertension, TG: Triglyceride, HDL: high-density lipoprotein cholesterol, MCVE: major cardiovascular events, MetS: metabolic syndrome, ALT: Alanine aminotransferase, LSM: liver stiffness measurement

 Metabolic syndrome and its component factors, except for raised blood pressure and WC, had a higher prevalence among patients with MAFLD in the non-Turkmen group (65.2% vs. 51.6%-60.9% in Turkmen men and women). All of the components of MetS were more prevalent in women than men in both ethnic groups, except for elevated triglycerides, which had the highest prevalence (64.2%) in non-Turkmen men and the lowest (48.0%) among Turkmen men.

 The current data also showed a higher rate of high ALT among patients with MAFLD (43.4%-56.9% vs.12.3%-33.0%) relative to non-MAFLD participants, with more proportion among women than men in both ethnic groups. The highest proportion of high liver fibrosis was observed in patients with MAFLD with female sex in non-Turkmen (18.7%) and male sex (17.9%) in the Turkmen population.

###  Sex as an Effect Modifier of the Association between Ethnicity and MAFLD

 In the sex-stratified analysis, after adjusting for other independent variables to determine the independent association of ethnicity with MAFLD, it was found that among men, non-Turkmen men maintained initially twice the odds of having MAFLD compared with Turkmen men (*P* = 0.008). On the other hand, the initially higher likelihood of MAFLD among non-Turkmen women decreased slightly. It became somewhat lower than that among Turkmen women, although this decrease did not reach statistical significance (Table 5).

**Table 5:** Crude and Adjusted OR for the relationship between MAFLD and ethnicity by sex

**Ethnicity**	**Women**			**Men**		
	**Crude OR**	**Adjusted OR**	***P*** ** value**	**Crude OR**	**Adjusted OR**	***P*** ** value**
Turkmen	0.83(0.63 - 1.11)	1.05(0.69 - 1.58)	0.826	0.59(0.45 - 0.80)	0.54 (0.34 - 0.86)	0.008^a^
Non-Turkmen	Reference	Reference		Reference	Reference	

Adjusted for demographic variables (age, education, smoking, opium, alcohol, physical activity status) and metabolic dysregulations (Waist ≥ 90 cm, BMI ≥ 25, IFG/Diabetes, Homa IR > 2.5, HTN, elevated TG, low HDL, MCVE, high ALT, LSM ≥ 7). ^a^ Adjusted OR statistically significant at *P* < 0.05.

###  Metabolic Disorders Associated with MAFLD among Men and Women by Ethnic Groups

 In the stratified adjusted multivariate analysis (Table 6), in all sex/ethnic strata, the OR of MAFLD with all metabolic disorders was modified. Among men, insulin resistance, high BMI, and high ALT maintained their independent association with MAFLD in both ethnic groups, while high TG remained only among the non-Turkmen group. Meanwhile, among women, MAFLD retained its association independently with increased WC, insulin resistance, IFG/T2DM, and high ALT in both ethnic groups but with high TG only among Turkmens. In the adjusted model, MAFLD showed the strongest OR with increased WC among women and insulin resistance among men in both ethnic groups.

**Table 6:** Crude and adjusted OR for the r relationship between MAFLD and metabolic disorders in sex/ethnic groups

	**Turkmen (n=854)**				**Non-Turkmen (n=760)**			
	**Crude OR **	***P*** ** value**	**Adjusted OR**	***P*** ** value**	**Crude OR **	***P*** ** value**	**Adjusted OR**	***P*** ** value**
BMI > 25								
Men	7.80 (4.21-14.44)	< 0.001^a^	3.19 (1.29 -7.85)	0.012^a^	10.09 (5.19-19.60)	< 0.001^a^	4.29 (1.65 -11.15)	0.003^a^
women	6.48 (3.12-13.47)	< 0.001^a^	2.21 (0.82 - 5.97)	0.116	20.21 (6.17-66.23)	< 0.001^a^	4.46 (1.02-19.54)	0.047^a^
Waist ≥ 90								
Men	12.52 (4.49 - 34.93)	< 0.001^a^	2.11 (0.50 - 8.79)	0.307	9.79 (4.37- 21.93)	< 0.001^a^	2.53 (0.75 - 8.44)	0.132
Women	21.50 (6.62- 69.76)	< 0.001^a^	6.10 (1.56 - 23.86)	0.009^a^	12.18 (5.09 - 29.13)	< 0.001^a^	4.80 (1.34 - 17.22)	0.016^a^
IFG /T2DM								
Men	2.32 (1.67- 3.21)	< 0.001^a^	1.60 (0.68 - 3.79)	0.279	2.69 (1.68 - 4.31)	< 0.001^a^	1.35 (0.68 - 2.68)	0.387
Women	4.77 (2.91- 7.82)	< 0.001^a^	2.28 (1.14 - 4.56)	0.020^a^	3.78 (2.29 - 6.22)	< 0.001^a^	1.94 (1.00 - 3.76)	0.049^a^
Homa-IR > 2.5								
Men	6.99 (4.38 -11.16)	< 0.001^a^	4.37 (2.27- 8.42)	< 0.001^a^	7.91 (4.96 -12.61)	< 0.001^a^	4.68 (2.56 - 8.55)	< 0.001^a^
Women	3.76 (2.49 - 5.68)	< 0.001^a^	2.36 (1.27- 3.93)	0.005^a^	6.58 (4.14 -10.46)	< 0.001^a^	3.81 (2.18 - 6.64)	< 0.001^a^
HTN								
Men	1.89 (1.13 - 3.16)	0.014^a^	1.45 (0.68 - 3.13)	0.328	1.17 (0.73 -1.87)	0.515	1.22 (0.62 - 2.39)	0.567
Women	1.31 (0.84 - 2.05)	0.232	0.49 (0.08 - 2.71)	0.413	1.74 (1.09 - 2.76)	0.019^a^	0.83 (0.44 -1.57)	0.573
High TG								
Men	2.59 (1.68 - 4.01)	< 0.001^a^	1.26 (0.65 - 2.43)	0.497	2.77 (1.82- 4.20)	0.002^a^	1.83 (1.01 - 3.34)	0.046^a^
Women	3.49 (2.31 - 5.29)	< 0.001^a^	2.09 (1.13 - 3.89)	0.019^a^	1.95 (1.28 - 2.97)	< 0.001^a^	1.43 (0.77- 2.64)	0.250
Low HDL								
Men	1.63 (0.88 - 3.01)	0.117	0.56 (0.22 - 1.45)	0.237	1.58 (0.94 - 2.65)	0.110	0.64 (0.29 - 1.42)	0.275
Women	2.26 (1.39 - 3.66)	0.001^a^	0.88 (0.41 - 1.88)	0.749	1.42 (0.92 - 2.20)	0.079	1.05 (0.55 - 2.01)	0.874
High ALT								
Men	5.68 (3.48 - 9.28)	< 0.001^a^	3.42 (1.68 - 6.93)	0.001^a^	5.29 (3.22 - 8.68)	< 0.001^a^	4.11 (2.14 -7.88)	< 0.001^a^
Women	3.57 (2.37- 5.38)	< 0.001^a^	3.02 (1.76 - 5.19)	< 0.001^a^	2.30 (1.69 - 3.13)	< 0.001^a^	2.46 (1.41- 4.29)	0.002^a^
LSM ≥ 7								
Men	2.78 (1.44 - 5.37)	0.002^a^	2.41 (0.91 - 6.40)	0.078	2.57 (1.29 - 5.09)	0.003^a^	2.32(0.20 - 0.63)	0.206
Women	1.77 (0.85 - 3.66)	0.123	1.13 (0.43 - 2.98)	0.809	3.26 (1.42 - 7.47)	0.005^a^	1.33(0.51- 3.05)	0.506

BMI: Body mass index, IFG/T2DM: impaired fasting glucose or type 2 diabetes mellitus, HOMA-IR: Homeostatic Model Assessment for Insulin resistance, HTN: hypertension, TG: Triglyceride, HDL: high-density lipoprotein cholesterol, ALT: Alanine aminotransferase, LSM: liver stiffness measurement. Adjusted for demographic variables (age, education, smoking, opium, alcohol, physical activity status) and metabolic dysregulations (waist ≥ 90 cm, BMI ≥ 25, IFG/Diabetes, Homa IR > 2.5, HTN, elevated TG, low HDL, MCVE, high ALT, LSM ≥ 7). ^a^ OR statistically significant at *P* < 0.05.

## Discussion

 The current study analyzed data from the PolyIran-liver database, which includes the urban population of the GCS study, to investigate the prevalence and characteristics of MAFLD, considering sex and ethnic differences in an Iranian multiethnic population for the first time. The study revealed a prevalence of 39.8% for MAFLD in the overall study population, highlighting disparities in sex and ethnicity.

 Noting that MAFLD is a newly defined condition, we only have one previous study comparing the prevalence and characteristics of MAFLD in Iran. This study focused on the 35-70-year-old population of Sabzevar and reported a rate of 22.8%, which is lower than our current findings.^25^ However, our findings align with the results of a systematic review that estimated a prevalence of 33.9% (95% CI: 26.4%-41.5%) for NAFLD in Iran, and additional studies have estimated a prevalence of 39.1% (95% CI: 36.3-42.1) for MAFLD in the US population and 31.7%-40.4% in the Liaoning province in China.^8,26,27^

 Our study discovered a negative association between MAFLD and Turkmen ethnicity in the bivariate analysis. After examining this association in the multivariable-adjusted model, in separate sex strata, Turkmen ethnicity retained a significant negative association with MAFLD only among men. Conversely, it exhibited a positive association among women, although no statistically significant differences were observed between ethnic groups in women. This finding confirmed a prior study conducted in the United States, which reported that sex acts as an effect modifier in the relationship between NAFLD and ethnicity.^28^

 The current study revealed a higher prevalence of MAFLD among women than men (45.8% vs. 34.1%). However, the female-to-male ratio varied across the ethnic groups. Turkmen women had a 1.5 times higher prevalence for MAFLD than men (43.8% vs. 28.6%), while the difference was less pronounced among non-Turkmen individuals (48.2% vs. 40.1%).

 The higher prevalence of MAFLD among women in our study contrasts with the majority of previous studies conducted in Iran, which reported a significantly higher prevalence of NAFLD among men.^8,29^ However, it aligns with two prior studies conducted in the northeastern provinces of Iran, which are geographically close to our study province.^25,30^

 The higher prevalence of MAFLD among women in this region can be attributed to the higher rates of obesity and MetS among them compared with men. Several previous results obtained from the Golestan cohort study have reported a higher rate of obesity and its comorbidities among women compared with men in the Golestan cohort and attributed it to the sedentary lifestyle of women in this region.^31-33^ Our study also showed a higher proportion of known risk factors for MAFLD, including increased WC, overweight/obesity, IFG/T2DM and MetS among women with MAFLD compared with men. These findings could be attributed to the sedentary lifestyle commonly observed in women over 50 years and their menopausal status. These factors are known to predispose women to higher rates of obesity and MetS in this age group.^34,35^

 Our data demonstrated the average rate of 43.5% for MetS among the overall population, with a higher proportion among the non-Turkmen general population (50.0% vs. 37.0% for Turkmen). Considering the proven link between metabolic characteristics and ethnicity,^36-38^ this finding can be attributed to the higher prevalence of MetS among patients with MAFLD in non-Turkmen ethnic group (65.2% for non-Turkmens vs. 55.9% for Turkmens).

 This study also revealed that while Turkmen men were significantly less likely than other groups to suffer from MetS or MAFLD, the prevalence of high liver fibrosis among them was nearly as high as that of non-Turkmen women, reaching the second highest levels. Considering that Turkmen men also exhibited the highest rates of lean MAFLD (MAFLD with BMI < 25), underlying genetic variations may contribute to the observed differences in MAFLD characteristics between Turkmen men and other groups. Investigating their susceptibility to lean MAFLD or poor liver outcomes requires conducting future genetic examinations and long-term prospective studies.^39-42^

 The adjusted multivariable logistic regression analysis of the present study identified differences in independent metabolic factors associated with MAFLD and ORs among different sex/ethnic groups. In explaining the reason, previous studies have suggested that sex-related unknown differences in the disease etiology and pathogenesis may be involved in differences in NAFLD/MAFLD characteristics between sexes.^28,35^ Furthermore, insulin resistance was recognized as the first strong independent association with MAFLD in men and the second in women in both ethnic groups. This finding aligns with previous studies that have suggested that insulin resistance is a crucial pathological factor in the interconnection between liver steatosis, abdominal obesity, and other metabolic abnormalities in patients with NAFLD/MAFLD.^43-45^ It also highlights the vital role of screening people at risk of insulin resistance and prevention and control through healthy lifestyle education.^46^

 The current study had several strengths and limitations. Strengths of this study included that it was the first to investigate the prevalence and characteristics of MAFLD, focusing on sex/ethnic differences in an Iranian population. In addition, this study was conducted on a large population-based sample that included comprehensive information on potentially relevant demographic and lifestyle factors collected in the Golestan cohort study.

 A major limitation was the inclusion of an urban population and age of over 50, which restricts the generalization of findings to rural and younger age groups. The second is the study’s cross-sectional design, which cannot determine either the cause-effect relation between MAFLD and its associated factors or its long-term outcomes. Thirdly, in the studied area, the non-Turkmen ethnic group consists of several ethnic groups, such as Fars, Turks, and Sistani, who had previously migrated to this region. Due to the smaller population size in this research, they were not analyzed and classified separately. However, future studies with a larger sample size are needed to investigate the characteristics of MAFLD separately in these subgroups. Additionally, this study did not investigate participants’ sleep patterns and dietary habits.

## Conclusion

 This study demonstrated a high prevalence of MAFLD with sex/ethnic disparity. Women with nearly one in two high-risk individuals were more likely to develop MAFLD regardless of ethnicity. Meanwhile, Turkmen men, despite the least likelihood of having MAFLD or MetS, were among the highest proportion of MAFLD patients with increased liver stiffness. Further investigations to identify the underlying factors that contribute to insulin resistance and obesity, particularly in women, as well as liver fibrosis and long-term outcomes of MAFLD, specifically among MAFLD patients of male sex and Turkmen ethnicity, will help to provide helpful information for developing more effective strategies to prevent and control of MAFLD and its the complications in this region.
